# Editorial: Bridging Membrane Biophysics to Microbiology: Innovating Towards New Peptide and Peptide-Based Antimicrobials

**DOI:** 10.3389/fmedt.2021.699154

**Published:** 2021-07-19

**Authors:** Lorenzo Stella, Sergey A. Akimov, Sattar Taheri-Araghi, Miguel A. R. B. Castanho

**Affiliations:** ^1^Department of Chemical Science and Technologies, University of Rome Tor Vergata, Rome, Italy; ^2^Laboratory of Bioelectrochemistry, Frumkin Institute of Physical Chemistry and Electrochemistry (RAS), Moscow, Russia; ^3^Department of Physics and Astronomy, California State University, Northridge, CA, United States; ^4^School of Medicine, Instituto de Medicina Molecular, University of Lisbon, Lisbon, Portugal

**Keywords:** lipid, peptide, drug, translation, bacteria

Molecular biophysicists and microbiologists working on antimicrobial peptides (AMPs) are traditionally worlds apart. While biophysicists prefer detailed structural and functional approaches in systems they have total control of, such as lipid vesicles, microbiologists trade the details of molecular interactions for the “biorealism” of working with live bacteria. These two types of studies seemed impossible to reconciliate, until now. In recent years, biophysical techniques started to be applied in the quantitative investigation of the interaction of AMPs with target and host cells, providing several novel insights in the mechanism of action of these molecules. The articles of this Research Topic provide a comprehensive overview of the current state-of the-art in this area.

Top researchers devoted to AMPs, translating molecular biophysical approaches to bacterial microbiology, have gathered in this Research Topic publication to (i) review how imaging and spectroscopy techniques can be used to unveil AMP action directly in live bacteria, and (ii) present the fundamentals of the mechanism of action of AMP obtained with such techniques, including the influence of intracellular factors, synergy, and cell density. Structure-function relationships and a vision for future applications of antimicrobial surfaces are also present.

Gelmi et al. present a general overview of the experimental approaches that provide information on peptide-cell interaction, including fluorescence and calorimetry to quantify peptide-cell association, circular dichroism, and NMR to investigate the secondary structure of cell-bound peptides, infrared and NMR spectroscopies to study peptide effects on membrane structure and dynamics, zeta potential experiments to clarify the electrostatic aspects of the interaction, microscopies, and cytofluorimetry to study peptide localization and effects on bacterial growth and perturbation of cell membranes, scattering techniques to study peptide effects on cell shape and photocrosslinking coupled with mass spectroscopy to identify potential protein targets.

Clayton reviews the applications of fluorescence microscopic techniques to follow in real time the main events involved in bacterial killing. These studies are starting to illustrate the complexities of peptide-cell interaction: for instance, they showed that the timescale of AMP effects on bacteria is orders of magnitude slower than that required for the perturbation of artificial membranes, identified preferred sites of attack in cell membranes and demonstrated that peptide sequestration by dead cells can protect the rest of the bacterial population from the action of AMPs.

Booth focuses on deuterium solid state NMR studies of intact bacteria treated with AMPs. This approach provides information on peptide-induced perturbation of cell-membranes. For instance, it showed that some AMPs can cause disruption of the lipid bilayer at peptide:lipid ratios lower than those needed for killing, suggesting that membrane disruption may not be the only mechanism by which they harm cells.

Separovic et al. address in-cell solid-state NMR studies of AMPs, too, providing an overview of the different molecules that can be investigated with this technique, including peptidoglycans, lipopolysaccharides, phospholipids, DNA and AMPs themselves. The power of this non-invasive technique is exemplified by a recent study, which revealed a peptide-induced disruption of the molecular packing of both bacterial membranes and DNA.

Benfield and Henriques focus on the experimental approaches that can determine the mechanism of action of AMPs, and particularly differentiate peptides perturbing cellular membranes from those that interact with intracellular targets. Disruption of membranes can be observed in cells, using fluorophores that detect membrane depolarization or accessibility of the intracellular space. By comparing membrane perturbation and cell viability, membranes can be confirmed or excluded as the main target of AMPs. Peptide uptake into bacterial cells can be investigated using flow cytometry and fluorescence microscopy. Finally, intracellular protein targets of AMPs can be identified using bacterial proteome microarrays.

Bechinger et al. analyze the crucial property of synergy in AMPs: often, two (or more) peptides together have a higher activity than the single components. The molecular mechanisms of this effect are still heatedly debated. For the synergism between magainin 2 and PGLa, different hypotheses have been put forward, involving direct interaction of the peptides or changes in peptide orientation, but experiments performed with lipid mixtures correctly mimicking bacterial membranes support lipid mediated effects that favor peptide/membrane binding for the AMP mixture. The authors also show that a major effect of a synergistic combination is to increase the steepness of bacterial killing as a function of peptide concentration. This finding is consistent with a previous study on other AMPs (1) and provides a novel perspective on AMP synergism.

Duong et al. also address synergism, both among different AMPs and between AMPs and other classes of antibacterials, including histones, which co-localize with AMPs in innate immunity components. Such synergies might arise if one antimicrobial agent favors pore formation by the other, or if it acts on intracellular targets that can be reached more easily after pore formation.

Marx et al. present original research that bridges the antimicrobial activity of two lactoferricin derivatives (LF11-215 and LF11-324) in *E. coli* and lipid vesicles. In particular, they determined both an upper limit on the number of surface-adsorbed peptides and total number of peptides partitioned into the bacteria These data indicate that 95–99% of LF11-215 and LF11-324 molecules accumulate inside cells, suggesting that these AMPs might act on intracellular targets.

Schefter et al., in their original work, discuss several parameters that influence AMP activity and selectivity: density of target and host cells, competitive peptide association to the two cell populations, and peptide sequestration by association to intracellular targets. The authors propose a biophysical model, based on chemical equilibria, leading to a prediction of the cell-density dependence of peptide activity and selectivity, and allowing the correct design and interpretation of selectivity measurements.

Strandberg et al. review the specific class of artificial AMPs constituted by sequences of alternating cationic and hydrophobic residues, such as [KL]_n_, or [RW]_n_. When bound to membranes, these peptides attain a beta-like structure. Longer peptides can also form beta-aggregates in solution. Medium length peptides of 8–10 amino acids appear to be the optimum compromise for a high activity and low toxicity.

Mullen et al. present their perspective on the use of peptides and other molecules to design antimicrobial surfaces. This is a view into the future of peptide-based products and technologies to fight bacterial threats, mainly biofilms, which are probably the next big challenge ahead for AMP developers.

After bridging molecular biophysics to microbiology, it is important to bridge these fields to bacterial biofilm biomedicine. AMPs are up to the challenge. Are we, too?

## Author Contributions

Manuscript content was discussed and decided by all authors. MC and LS drafted the manuscript. All authors revised the manuscript.

## Conflict of Interest

The authors declare that the research was conducted in the absence of any commercial or financial relationships that could be construed as a potential conflict of interest.
